# Microbiologically Induced Concrete Corrosion: A Concise Review of Assessment Methods, Effects, and Corrosion-Resistant Coating Materials

**DOI:** 10.3390/ma15124279

**Published:** 2022-06-16

**Authors:** Bhavesh Chaudhari, Biranchi Panda, Branko Šavija, Suvash Chandra Paul

**Affiliations:** 1 Centre for Intelligent Cyber Physical Systems, Indian Institute of Technology Guwahati, Assam 781039, India; cbhavesh97@rnd.iitg.ac.in; 2 Department of Mechanical Engineering, Indian Institute of Technology Guwahati, Assam 781039, India; 3 Microlab, Faculty of Civil Engineering and Geosciences, Delft University of Technology, 2628 CN Delft, The Netherlands; b.savija@tudelft.nl; 4 Department of Civil Engineering, International University of Business Agriculture and Technology, Dhaka 1230, Bangladesh; suvashpl@iubat.edu

**Keywords:** microbiologically induced corrosion, corrosion-resistant coatings, sewage pollution, environmental risks

## Abstract

Microbiologically induced concrete corrosion (in wastewater pipes) occurs mainly because of the diffusion of aggressive solutions and in situ production of sulfuric acid by microorganisms. The prevention of concrete biocorrosion usually requires modification of the mix design or the application of corrosion-resistant coatings, which requires a fundamental understanding of the corrosion process. In this regard, a state-of-the-art review on the subject is presented in this paper, which firstly details the mechanism of microbial deterioration, followed by assessment methods to characterize biocorrosion and its effects on concrete properties. Different types of corrosion-resistant coatings are also reviewed to prevent biocorrosion in concrete sewer and waste-water pipes. At the end, concluding remarks, research gaps, and future needs are discussed, which will help to overcome the challenges and possible environmental risks associated with biocorrosion.

## 1. Introduction

Large water and wastewater treatment plants, conduits, and pipelines are most widely constructed using concrete. This is due to its longevity, local availability, ease of use, and low cost [1]. Although concrete is one of the most suitable construction materials for many applications, it has limitations in severe environments such as sewerage systems. For maintaining expected sanitary standards in modern society, an efficient, safe, and cost-effective wastewater collection and transport system is required [2]. If the network system is insufficient or lacks in operation, it can cause the spread of infectious diseases and contamination of drinking water, especially in developing countries [3].

The total length of the sewage networks in countries such as the US, the UK, Japan, Germany, and China is 10 times more than the circumference of the earth [4]. Sewage pipes mostly carry organic and inorganic substances, which may be corrosive, resulting in the degradation of concrete [5]. Microbially induced concrete corrosion (MICC) is one of the main processes for the degradation of concrete worldwide causing high economic expenses along with severe health and environmental concerns [6,7,8,9]. Microbially induced corrosion (MIC) can be defined as the process in which biological agents (live organisms) cause changes in the material properties leading to the structural lowering in quality or value. This biodegradation of concrete significantly affects the durability of the infrastructure by reducing its lifespan to 30 to 50 years, from a designed life of 100 years, depending upon the severity of the environment [10]. In addition to economic losses, the MICC produces hazardous gases, such as hydrogen sulfide (H_2_S), carbon dioxide (CO_2_), ammonia (NH_3_), methane (CH_4_), and other volatile organic compounds (VOCs), representing a severe health risk for workers and operators of wastewater systems [11,12]. By considering the above economic losses and health issues, it is necessary to find long-term sustainable solutions for biocorrosion of concrete structures.

There have been several reviews published on MIC focusing on the aspects such as the use of nanotechnology [13], advances in geopolymer on wastewater applications [14], MIC of metals [15], marine environment corrosion [16], and microorganisms present in the sewer environment [8,17]. However, to the best of the authors’ knowledge, very few studies have been carried out on MIC of concrete exposed to sewer environments with emphasis on different coating materials to protect concrete from corrosion. This work aims to critically summarize the existing assessment methods to characterize MIC and its effects. MIC mechanisms and different coating materials having resistance to biogenic corrosion are also reviewed. It is expected that this review will be useful for solving the challenges and environmental risks associated with MICC and will help readers to clearly understand the pros and cons of all the available assessment methods so that they can choose the correct coating materials either for real-life applications or for further studies based on their experimental results. 

## 2. Background

In 1945, Parker [18] discovered the presence of bacteria in the corrosion process, and since then, the study of microbiologically induced corrosion has started. Since then, various researchers have made efforts to understand the exact mechanism behind it. According to [14], MICC is a complex process that requires an interdisciplinary approach between the fields of civil and chemical engineering, microbiology, hydrochemistry, mineralogy, as well as environmental sciences.

**Figure 1:**
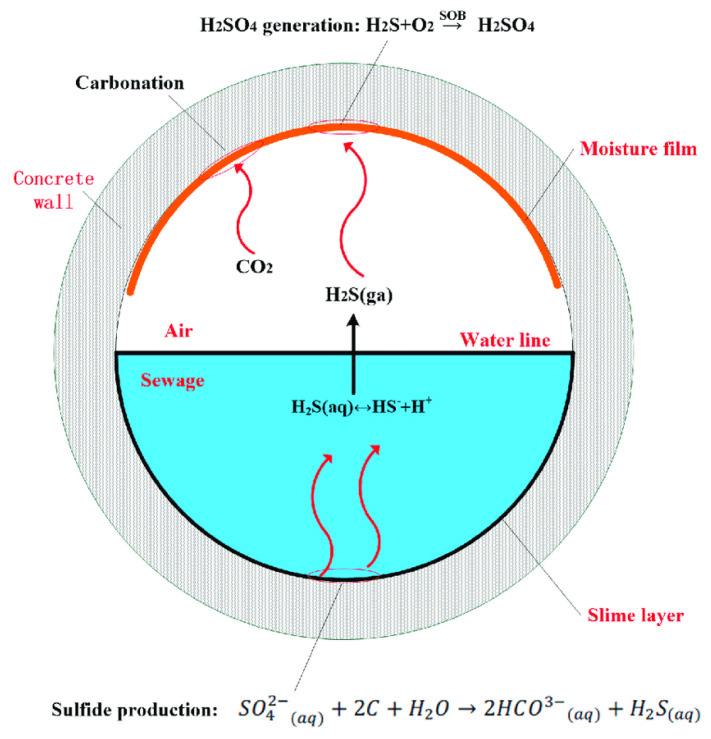
The sulfur cycle in the sewer system [31].

The complex, three-stage microbiological chemical process of MICC starts with the reduction of sulfates present in wastewater into hydrogen sulfide (H_2_S) by sulfate-reducing bacteria (SRB), e.g., Desulfovibrio and Desulfomaculum [19,20,21]. This reduction occurs in the anaerobic environment at the bottom part of the sewer (Figure 1). Initially, the surface pH of the freshly prepared concrete is approximately 12 to 13 depending on the type of concrete. This initial pH is reduced to around 9 because of the acidification of H_2_S gas to thiosulfate and polythionic acid along with abiotic neutralization by carbonation, making the environment suitable for the growth of sulfur-oxidizing bacteria (SOB) [21,22,23]. The turbulence in the sewage and decrease in surface pH cause H_2_S to escape into the sewer atmosphere and adhere to the concrete [19]. 

The second stage of MICC is initiated by the growth of microbiological colonies on the concrete surface. Initially, sulfur-oxidizing bacteria grow on the surface of the concrete, which produces sulfur-based chemicals (sulfur and polythionic acid), further reducing the pH of the surface [24]. From this point, the corrosion of the concrete matrix begins. Mainly, thiosulfate (S_2_O_3_^2−^) and sulfur (S_0_) act as intermediates for the oxidation of H_2_S to sulfate (SO_4_^2−^). These intermediates act as an energy source for many thiobacilli SOB [14]. Acidithiobacillus thiooxidans, Acidithiobacillus ferrooxidans, Thiomonas intermedia, and Halothiobacillus neapolitanus are some of the commonly found SOB, out of which H. neapolitanus, T. intermedia, and A. thiooxidans are the most aggressive strains of SOB. These strains of SOB further deteriorate the concrete, thereby reducing the pH [25,26,27]. 

**Figure 2:**
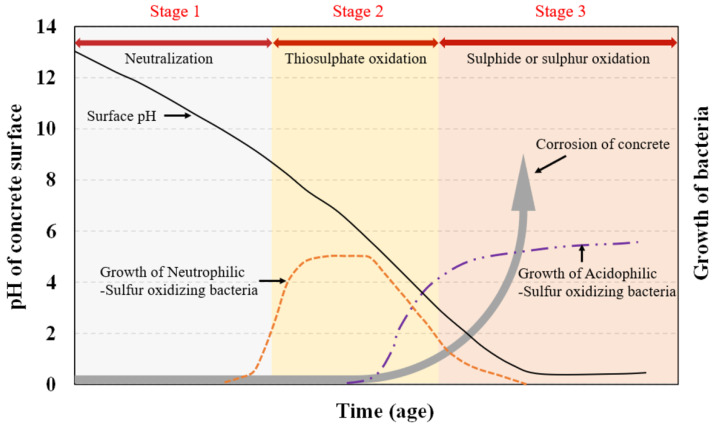
Stages of biocorrosion in concrete [33].

In the last stage of MICC, the metabolization of sulfur and thiosulfate into sulfuric acid (H_2_SO_4_) takes place by acidophilic SOB [28,29]. This causes an additional reduction in the pH of concrete, especially on the surface, reducing it to around 2. The developed sulfuric acid reacts with the calcium hydroxide (CH) present in the concrete, leading to the formation of gypsum (CaSO_4_.2H_2_O). Subsequently, gypsum reacts with calcium aluminate hydrate (C_3_A) and forms ettringite (3CaO.Al_2_O_3_.3CaSO_4_.32H_2_O) [19,30]. Because of the ettringite formation, the volume of the material expands by up to 700%, which causes exfoliation and cracking in the concrete microstructure [9,20,31]. These stages of MICC were initially summarized and adopted by [29,32] (Figure 2).

## 3. Biocorrosion Assessment Methods in Concrete

Since 1945, when Parker [18] reported the presence of bacteria in the corrosion process, numerous studies have been carried out regarding various methods to study MICC due to the lack of standardized testing methods. These methods can broadly be categorized into three groups: chemical tests, laboratory simulation tests, and in situ tests. In this section, these three test methods are discussed in detail.

### 3.1. Chemical Tests

Chemical tests can be performed either by keeping the rate of degradation the same, as observed in reality, or by accelerating the process. Realistic concentrations of the aggressive acids/salts are used in the former case, whereas in the latter, the rate of degradation is increased either by increasing the concentration of the acid, raising the temperature, or increasing the contact surface area/volume ratio [34,35,36]. Though accelerated tests are more widely used [37], they may cause problems such as a change in the attack mechanism [38]. In general, sulfate solutions and sulfuric acid solutions are mainly used for studying biogenic sulfuric acid corrosion. However, Monteny et al. concluded in their research that for the chemical part of the corrosion, sulfuric acid should be used instead of sulfate solutions [20].

**Figure 3:**
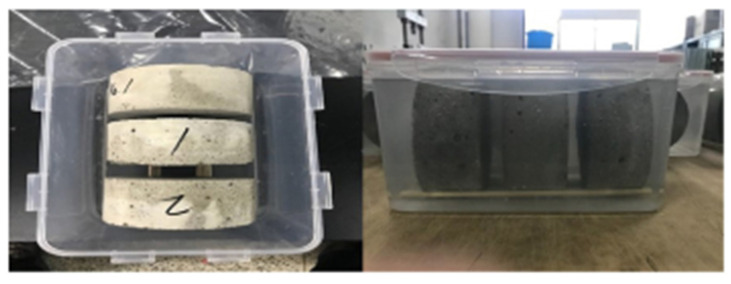
Sulfuric acid immersion test [39].

In chemical tests, prismatic mortar samples of dimensions varying from 25 × 25 × 250 mm to 40 × 40 × 200 mm, cylinder height from 50 to 150 mm, and cube dimensions from 50 to 100 mm are prepared. These samples are then immersed in the aggressive solutions of either sodium or magnesium sulfate or sulfuric acid [20,39] (Figure 3). The concentrations of sulfate and sulfuric acid solutions vary from 5–10% and 1–10%, respectively [39,40,41]. While samples are immersed, the pH of the solutions is kept constant either by automatic titration or by replenishing it at regular intervals. The duration of sample immersion can range from 1 to 3 years in the case of sulfate solutions and from 7 days to 6 months in the case of sulfuric acid solutions [20,42]. Then, the samples are taken out and the performance of concrete is evaluated based on parameters such as strength loss, weight loss, penetration index, change in geometry, surface morphology, etc. [20,39,43].

The advantage of chemical tests is that they are relatively simple, and the testing time is much shorter compared to biological tests [43]. However, while chemical tests mimic the last stage of MICC, i.e., sulfuric acid attack, they do not reflect the biological aspects of MICC [44,45,46]. Additionally, results obtained from chemical tests are highly dependent on various factors, such as concentration of acid, duration of immersion, sample conditioning, and exposed sample area, which makes it difficult to interpret the results [47]. A summary of different chemical tests reported in the literature is given in Table 1.

### 3.2. Laboratory Simulation Tests

As discussed in the above section, MICC is a complex and often slow process (1 mm/year to 5 mm/year) [51]. Therefore, the investigation of the performance of different materials against biogenic corrosion takes several years, as the process involves chemical as well as mechanical aspects. To address this problem, various researchers tried to simulate the corrosion as it occurs in situ. By creating favorable conditions for bacteria (temperature, nutrients), the rate of corrosion can be increased.

**Figure 4:**
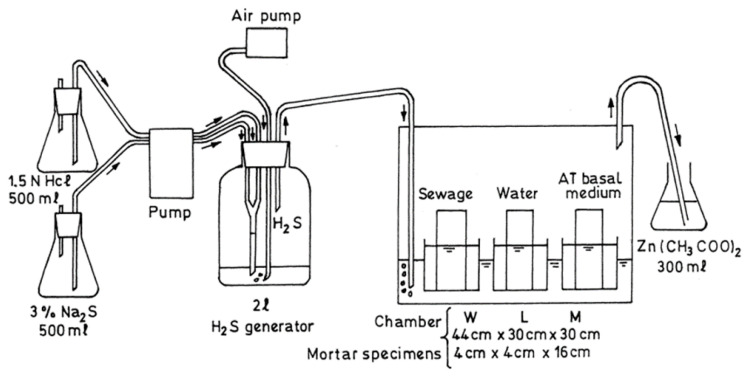
Simulation chamber developed by [52].

Mori et al. [52] developed a simulation chamber to investigate the effects of nutrients on the corrosion of concrete. They used a much higher concentration of H_2_S (400 ppm) in the chamber as compared to that observed on site. Mortar samples with dimensions of 4 × 4 × 16 cm were placed in a solution containing nutrients and minerals desirable for bacterial growth without thiosulfate. The duration of the experiment was 6 months. Furthermore, during the first two months, the samples were inoculated with T. thiooxidans every two weeks. A scanning electron microscope was used for the investigation of corroded samples, and the reduction in cross section of specimens was used to determine the corrosion rate. Additionally, by plate counting of the bacteria, the number of T. thiooxidans was determined. The authors concluded that to cause the maximum corrosion rate, nutrients and oxygen must be present. The simulation chamber developed by [51] is shown in Figure 4.

**Figure 5:**
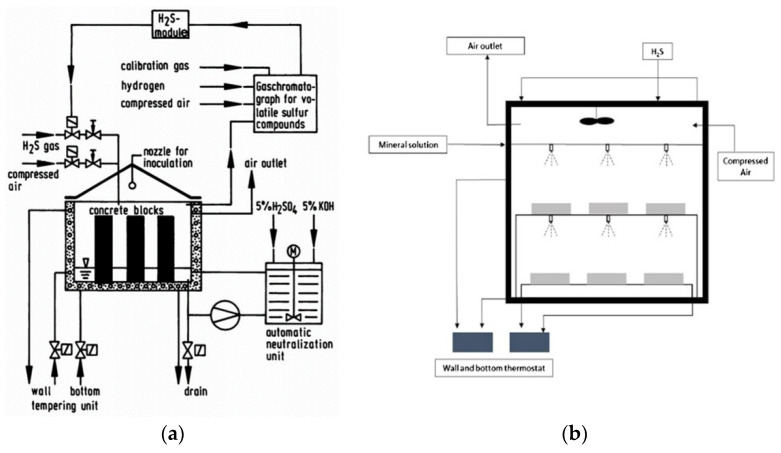
(**a**) Original Hamburg chamber [54]; (**b**) Hamburg chamber modified by [53].

Ehrich et al. [53] modified the Hamburg chamber [54,55], which had a corrosion rate 8 times faster than in situ. They used mortar samples with dimensions 2 × 2 × 2 cm instead of concrete samples. Also, the concentration of hydrogen sulfide (H_2_S) was controlled at 10 +/− 5 ppm along with relative humidity higher than 98%. It was observed that the corrosion rate in the modified chamber was 24 times faster than in situ. According to Scrivener et al. [46], the Hamburg chamber is the most representative laboratory test for studying MICC, and the setup of the chamber is shown in Figure 5a,b.

**Figure 6:**
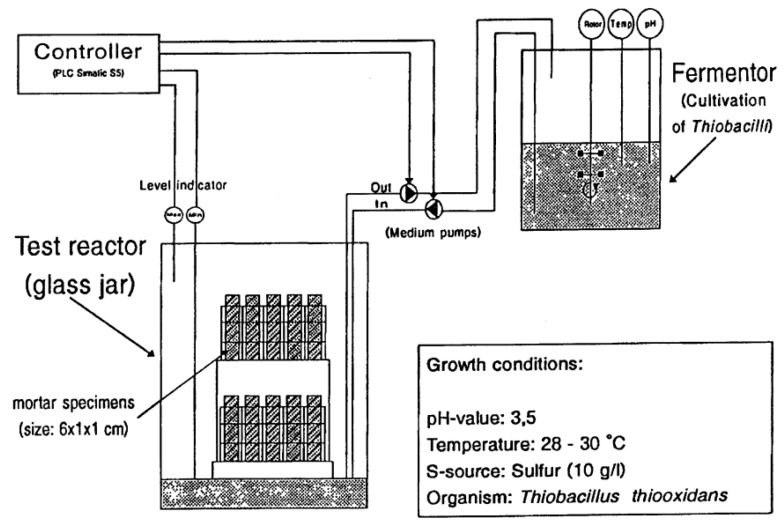
Heidelberg chamber for biogenic acid corrosion [56].

The Research, Development, and Consulting Department of Heidelberg University developed another simulation chamber [56,57]. In this chamber, the time required to investigate the resistance of materials against biogenic corrosion was reduced to 3–5 months. As shown in Figure 6, the chamber consists of the growth and reaction parts. The test specimens were kept in the reactor part of a bioreactor made of glass, whereas T thiooxidans was cultivated at optimum conditions in the growth part. A warm (28–30 °C) and humid environment was maintained in the reactor. Bacteria solution was sprayed on the specimens of dimensions 10 × 10 × 60 mm for 5 min every hour. The performance of concrete against corrosion was measured by using the weight loss of the test specimens and by determining the cell density on the surface of the specimens. Some researchers [58,59], argue that, although bacteria are involved in the process, it is still a pure acid test because of the 55 min gap. The corrosion rate in the chamber as compared to in situ is still not clear. The test setup for the Heidelberg chamber is shown in Figure 6.

Researchers from Ghent University developed a simulation chamber using the cyclic method [19]. The main reason for incorporating the cyclic method in the simulation chamber was to simulate the worst site conditions in a simple test. Each cycle consists of 4 steps: (a) exposure to H_2_S of 250 ppm for 3 days (b) immersion in the solution containing Thiobacillus bacteria for 10 days (c) rinsing by distilled water for 2 days and (d) drying for 2 days. Saucier et al. [59] identified that such testing conditions are far from reality, and due to 10 days of immersion in an acidic environment, it can be considered a pure acid attack irrespective of the bacterial presence. Similar to the Heidelberg chamber, the corrosion rate in the chamber as compared to in situ conditions is not known.

**Figure 7:**
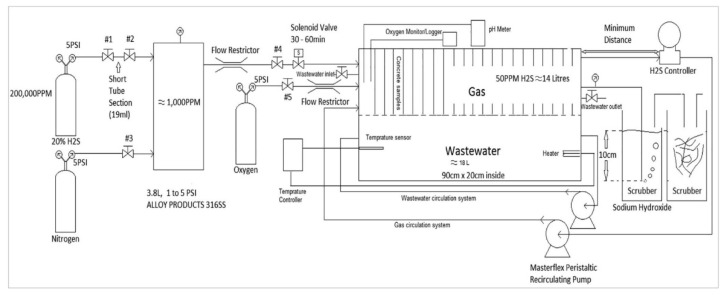

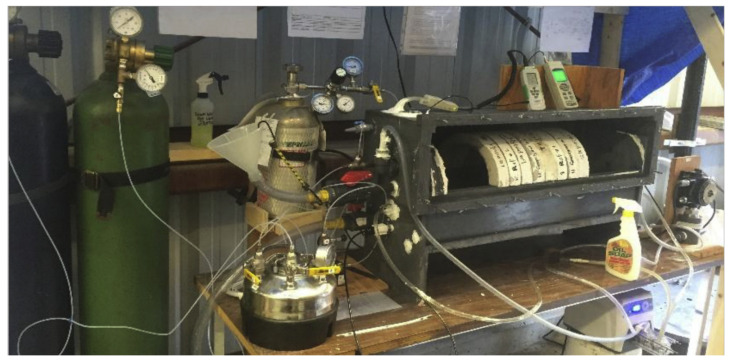
Chamber for simulating concrete biocorrosion [1].

Recently, Roghanian et al. [60] developed a chamber to obtain a controlled environment to simulate the real sewer conditions. To replicate the gravity conditions in sewers, a reactor of dimensions 90 × 20 × 10 cm of 10 mm thick poly (vinyl chloride) panels with free water surface was constructed. The main components of the reactor, shown in Figure 7, were an intermediate container; the main chamber containing concrete specimens (arch-shaped); tanks of H_2_S, nitrogen, and oxygen; a wastewater circulation system; and an air circulation system. H_2_S and nitrogen were injected from tanks into intermediate containers whereas oxygen was injected directly into the chamber. The concentration of H_2_S and oxygen was maintained at around 50 ppm and 15% for all corrosion cycles, which were performed using the H_2_S gas monitor and oxygen meter/logger, respectively. The intermediate tank was refilled manually when the pressure dropped from 5 psi to 1 psi. The temperature of 28 °C +/− 1 °C, relative humidity of 85 +/− 5%, and oxygen level of 15 +/− 5% were kept constant during the entire test for optimum growth of bacteria. After reaching a stable pH, approximately two thirds of the wastewater were replaced by fresh wastewater every 2 to 3 weeks. To study the reliability of the chamber, arch-shaped concrete samples were placed in the chamber for a 6-month duration, and parameters like flexural strength loss, pH variation, and surface morphology were evaluated. This chamber has the potential to act as the most representative laboratory test for studying MICC, as the authors have compared the in-situ conditions with simulated conditions in detail.

A review of various test methods that simulate real sewer conditions was recently published by Madraszewski et al. [61]. They discussed the important types of concrete durability simulation tests, the most used testing parameters of microbiological tests, provided a comparison study of the degree of acceleration of each simulation test, and also highlighted the importance of the application of bacteria and nutrients during testing. They concluded that the laboratory simulation tests more accurately reflect the real sewer conditions than chemical tests and also better explain the entire MICC process. They also found that the Ghent setup is the best method based on the comparison of conditions applied in the method to the conditions occurring in real sewers.

### 3.3. In Situ Tests

Laboratory simulation tests take much less time compared to the actual site’s corrosion process, but they are never completely satisfactory for durability problems, as it is always difficult to reproduce all the natural conditions and interactions under artificial conditions in the laboratory [20]. To overcome these limitations, in situ exposure tests come into the picture, which account for all the factors and interactions taking place in the real biogenic corrosion process. Various researchers have performed in situ tests in two ways; (i) preparing samples and then keeping them in sewers at a location of interest, or (ii) collecting samples from sewers where corrosion had taken place. Although in situ tests provide the most reliable results for biogenic corrosion, care should be taken while applying the conditions to other sites, as it involves various parameters which may or may not be the same at different sites. 

Mori et al. [52] made mortar specimens of dimensions 40 × 40 × 160 mm with Portland cement and a water/cement ratio of 0.65. To perform in situ exposure tests, they kept the samples in a highly corroded sewer pipe to study the effects of biogenic sulfuric acid for 8 months in total. The H_2_S concentration and the temperature in the pipe were in the range of 5 to 400 ppm and 10 °C to 30 °C respectively. The authors observed a corrosion rate of 5.7 mm/year.

For prediction of the likely present and future internal corrosion of the sewer pipes, [62] developed rational mathematical models based on field observations. They used two different concretes: (i) new coupons cut from a newly manufactured 1.2 m ID spun-cast standard reinforced concrete sewer pipe and (ii) old coupons cut from a 70-year-old sewer pipe that carried domestic, industrial, and trade waste in Perth, Australia. The samples were cut to 100 mm (nominal) cubes and care was taken that the previously corroded face remains undisturbed while cutting and handling. The samples were exposed for around 31 months in an aggressive sewer environment with a temperature of 26 °C, relative humidity 98%, and an H_2_S concentration of 79 ppm. After the exposure, the samples were inspected under an optical microscope, and changes in surface chemistry/mineralogy and depth of the corrosion product layer were determined. Based on the field observations, the authors concluded that bilinear models can be applied for corrosion loss, with negligible corrosion in the early period and then at a constant rate after the initiation. Later, Wells et al. [63] extended the study to model concrete deterioration in sewers based on theory and field observations. This time, they kept the two concretes (new and old) in six different sewers in Australia for 48 months and found that the corrosion losses at each site followed the bilinear trend proposed in an earlier study. The authors also developed the first pass model to find the rate of concrete sewer pipe corrosion using the average sewer temperature, H_2_S concentration, and humidity as known variables. Model predictions were found in good agreement after testing against reported corrosion rates.

## 4. Effects of Biocorrosion on Concrete Properties

Biocorrosion changes the concrete physical appearance as well as its internal structure. Physical changes can occur in terms of change in geometry, formation of surface cracks, surface material removal, or color change. Also, biodeterioration can cause microcrack formation, permeable gaps, or changes in the chemical composition of the material. This ultimately results in strength reduction and a decrease in the service of the structures. The results obtained by various researchers to study the effects of biocorrosion are discussed in this section. 

### 4.1. Visual Changes

**Figure 8:**
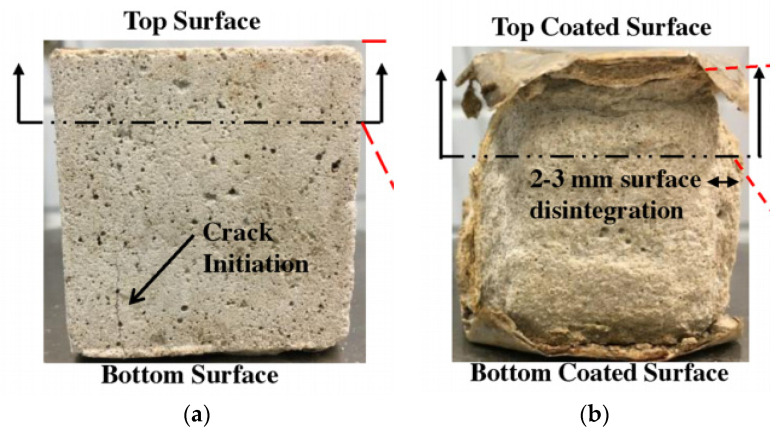
Visual assessment of geopolymer mortar after two years of exposure [64]. (**a**) FA-GPm; (**b**) SRPC.

After two years of exposure to the natural sewer environment, the samples of fly-ash-based geopolymer mortar (FA-GPm) and sulfate-resistant Portland cement mortar (SRPCm) showed surface degradation. SRPCm showed major degradation and a loss of 2–3 mm from the surface, whereas FA-GPm showed a smooth cubical surface with minor crack propagation near the edges [64]. The crack initiation and surface degradation of the samples are shown in Figure 8. House et al. [50] observed that most of the samples with dimensions of 38 × 38 × 200 mm prepared with different mixtures showed a 10 to 12 mm reduction in width. Monteny et al. [45] performed microbiological tests on prisms (20 × 20 × 50 mm) and chemical tests on cylinders (230 mm diameter and 70 mm height). At the end of the tests, the largest cumulative loss (after four cycles) in height of prisms in all mixtures was 0.76 mm, whereas up to a 0.6 mm decrease in average radius was observed in cylinders.

### 4.2. Microscopic Changes

**Figure 9:**
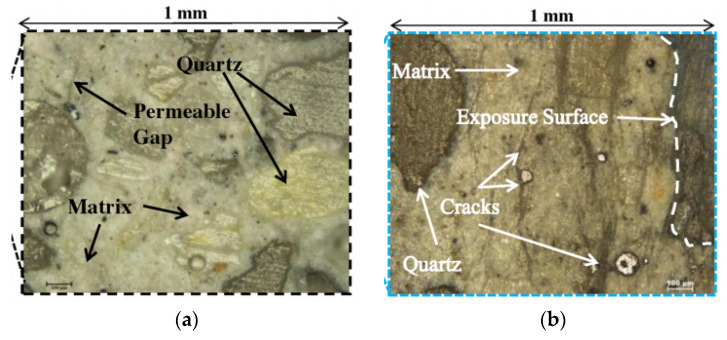
Optical microscopic image analysis of geopolymer after two years of exposure [64]. (**a**) FA-GPm; (**b**) SRPC.

Due to biogenic corrosion for 180 days, [1] observed that dissolved and ionized calcium penetrated the network of pores and leached from the system. In the corroded samples, the amount of sulfur increased significantly and zinc along with calcium almost leached out completely from the system. In [64], the authors performed optical microscope imagery on the specimens collected near the exposure surface and observed signs of physical deterioration such as cracks, gaps, and aggregate matrix debonding in both fly-ash-based geopolymer mortar (FA-GPm) and sulfate-resistant Portland cement (SRPC) mortars (as shown in Figure 9), which indicated the dissociation of the matrix. Due to a biogenic acid attack, permeable gaps seen within the matrix may be due to the breakage of the aluminosilicate matrix. The authors of [5] found that the hydration product of corrosion-resistant mortar (CY) was loosed and porous after 90 days of corrosion in sulfuric acid solution, which resulted in significant decomposition and destruction.

### 4.3. pH Variations

As discussed in Section 2, the surface pH of the concrete drops to 10–9 and 5–4 after stages I and II, respectively. A similar trend was observed by Khan et al. [64]; an average drop of 3.4 and 4.25 over 12 months was seen FA-GPm and SRPCm after stage I. Additionally, in a study carried out by [1], the pH of the samples with coatings of a blended mix of geopolymer and magnesium phosphate, geopolymer, and cement reduced to 6, 5, and 2, respectively, after 180 days of exposure to biocorrosion.

### 4.4. Mass Loss

Uncoated concrete specimens displayed weight loss of over 2% after immersion in 3% sulfuric acid for 7 days [48]. For FA-GPm and SRPCm, the average mass loss was observed to be 7.4 and 19.2%. The porosity of the concrete increased from 18.4 to 27.9% in the case of FA-GPm and from 17.8 to 23.4% in the case of SRPCm [65]. The reference mortar used by Zhang et al. [65] displayed a mass loss rate of 3%, mainly due to water evaporation, after 28 days, which was slightly decreased by adding silica fume. When specimens were immersed in sulfuric acid with a pH of 0.5, [49] a change in mass from 25 to 50%, based on the mixture type, was observed after 56 days.

### 4.5. Strength Loss

The cement mortar samples without any coating showed a decrease in flexural strength of 73%, and corresponding deflection increased by 50% after keeping the samples in a biocorrosion chamber for 180 days (1). In a study by [64], the failure load of FA-GP mortar dropped from 130 KN to 56.7 KN (compressive strength from 50.5 MPa to 22.3 MPa) and from 127 KN to 33.7 KN (compressive strength from 49.3 MPa to 27.9 MPa) in the case of SRPC mortar. Almost half of the compressive strength was lost for mortars with corrosion-resistant admixture (CY) when exposed to sulfuric acid with a pH of 2 for 90 days [5]. Based on the mixture type, the relative dynamic elastic modulus (RDEM) of the specimens decreased to 80% to 60% after 112 days of immersion in sulfuric acid with a pH of 0.8 [50]. The compression test carried out on specimens classified into three groups with a total of 24 mixtures with varying proportions by [66] showed an increase from 3.1% to 34% after immersion in sulfuric acid (5% concentration) to 12 weeks. Though most researchers observed a strength decrease after biogenic corrosion, Harbulakova et al. [65] found an increase in the compressive strength of the concrete samples by 68% and 17% after 12 and 18 months of exposure, respectively. The use of high-performance concrete and continued hydration during exposure to wastewater could be the reason for an increase in the strength of concrete. The effect of biocorrosion on strength reported by various researchers is summarized below (Table 2).

## 5. Corrosion-Resistant Coating Materials

Concrete infrastructure exposed to corrosive environments can be protected by modifying concrete mix, replacing concrete with corrosion-resistant materials, and applying a corrosion-resistant coating layer on the inner side of the sewage pipe [68,69]. Intensive research has been carried out to investigate the fundamental corrosion process. However, there is no sustainable material that can entirely withstand extremely aggressive and corrosive sewer environments [58,70,71,72]. Acid and/or bacterial penetration may occur on the coating layers, which may result in a corroded substrate material behind the liner and ultimately destroy the bond. Blistering or coating failure is also possible in some cases when the coating impairs the breathability of concrete [72]. Other issues, such as cost, compatibility with parent material, corrosion, short lifetime, and toxicity may also be associated with the coating layers [73]. However, various attempts have been made to develop novel coating materials to mitigate these limitations, and this section describes some of the coating materials developed to prevent biocorrosion. 

**Figure 10:**
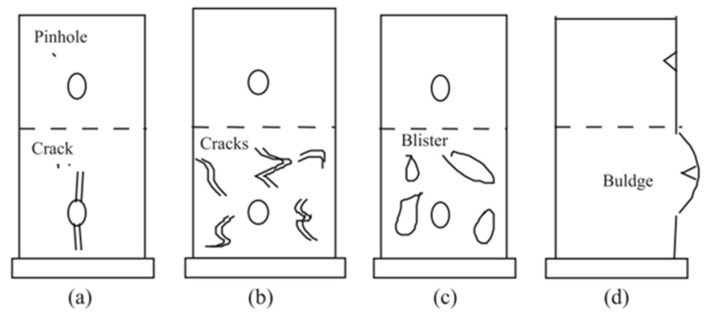
General failure types in coated concrete specimens with pinholes [49].

Vipulanandan and Liu [49] used two polyurethane-based coatings with slightly different properties, such as density, hardness, thickness, etc., to protect concrete pipes from corrosion. The performance of the coatings was evaluated under a 3% sulfuric acid solution (pH = 0.45; representing the worst reported condition in the wastewater system) environment for more than five years. The combination of a full-scale hydrostatic test, bonding test, and chemical resistance test was performed for evaluation. The hydrostatic test results showed the overall rating of the coating as “pass” and “satisfactory” on dry and wet application conditions, respectively. Contradictory results were seen, as one coating had very good bonding strength on the dry concrete surface but low bonding strength on the wet surface, whereas the other coating had very low bonding strength on a dry surface and was better on the wet surface. Both coatings performed extremely well in the chemical resistance test, as no failure was observed either coating after five years of immersion. The authors identified the failure types of concrete specimens as cracking of coating starting from the pinhole, or on the surface, and blistering at the pinhole (Figure 10).

**Figure 11:**
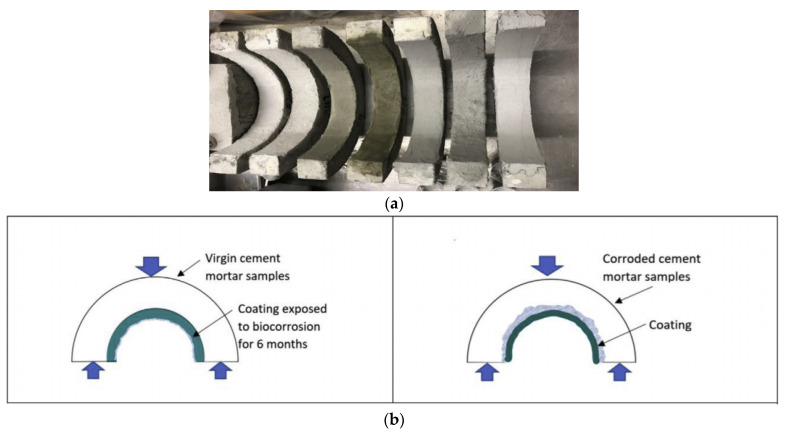
(**a**) arch-shaped samples with applied coatings (**b**) coatings placed on virgin (left) and corroded (right) samples [1].

**Figure 12:**
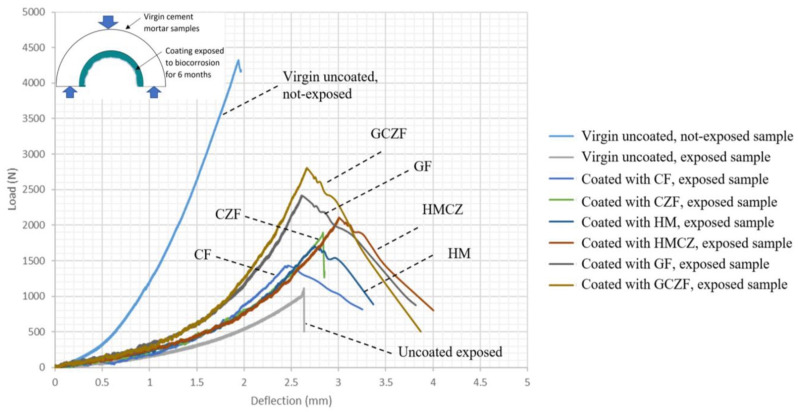
Effect of corrosion on the ultimate load-bearing capacity of different virgin coated samples [1].

Roghanian et al. [1] evaluated the performance of eight coatings developed from three base binders, i.e., cement mortar, geopolymer, and a blended mix of geopolymer and magnesium phosphate. Two broad mechanisms of directly adding zinc oxide as an antibacterial agent to the coating material and mixing zinc-doped clay particles with the coating were considered. Thirty arch-shaped cement mortar samples were cast to represent the upper half portion of the sewage pipe (Figure 11a). After curing the samples, their surfaces were prepared, and a 6 mm coating was applied. Again, after the curing period, samples were kept in an accelerated biocorrosion chamber developed by the authors for a six-month duration. The results showed that the surface pH of the cement-based samples reduced to an average of 2 by the end of the corrosion cycle, while geopolymer and blended samples showed pH values of 5 and 6, respectively. All three coating materials significantly improved flexural strength compared to uncoated samples, with geopolymer having the highest. Strength loss after corrosion increased to 35% for multiphase composite coating and geopolymer coating, which was much less compared to cement samples (52%) and uncoated samples (73%) (Figure 12). A similar trend was observed in the pull-off test, i.e., cement-based samples showed the lowest bond strength, followed by blended samples and geopolymer. Additionally, after applying geopolymer coating to corroded cement mortar samples, the pipe restored its strength by an average of 40%, which shows that geopolymer samples can be effectively used to protect not only virgin pipes, but also corroded pipes (Figure 11b).

**Figure 13:**
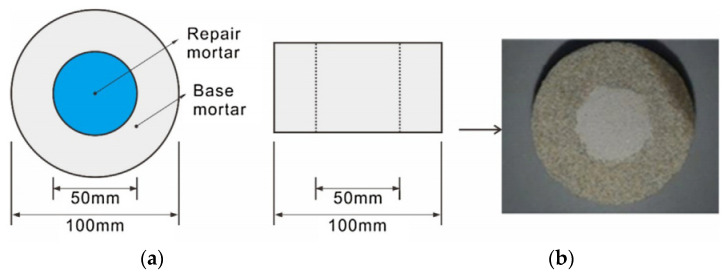
Test specimen details (**a**) top view, (**b**) side view [39].

**Figure 14:**
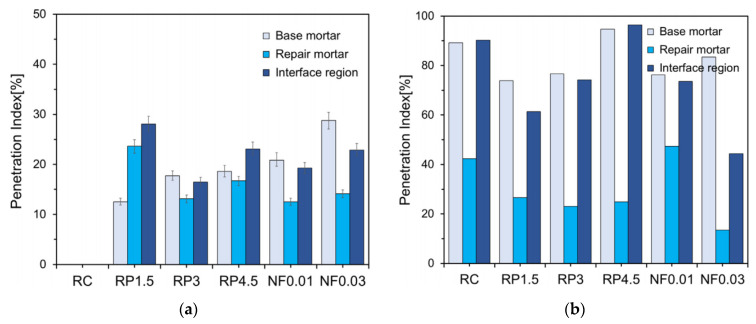
Penetration indices of the accelerated water tightness test after (**a**) sulfuric acid immersion and (**b**) freeze–thaw cycles [39].

Coatings of resin powder composed of polyvinyl acetate (PVA) and nylon fibers were developed by Chang et al. [39]. Resin powder with PVA enhances the acid resistance, watertightness, and adhesion of the coating to the parent material, whereas nylon fibers increase tensile strength, shrinkage resistance, and chemical stability. Circular hollow cylinders were cast from base mortar and were filled with repair mortar after curing (Figure 13). Three tests were performed: an accelerated test on the watertightness of the interface, an accelerated test on the watertightness of the interface after 10% sulfuric acid immersion for 7 days, and an accelerated test on the watertightness of the interface after 100 freeze–thaw cycles. The penetration index (PI) was calculated by dividing penetrated area by the total area. Figure 14 shows the performance of the coatings based on the PI of the accelerated water tightness test after sulfuric acid immersion and freeze-thaw cycles. Based on the test results, the authors recommended 4.5% resin powder coating without fiber under moderate environmental conditions. For severe environments demanding high sulfur and freeze–thaw resistance, a combination of PVA resin powder and nylon fiber was recommended.

**Figure 15:**
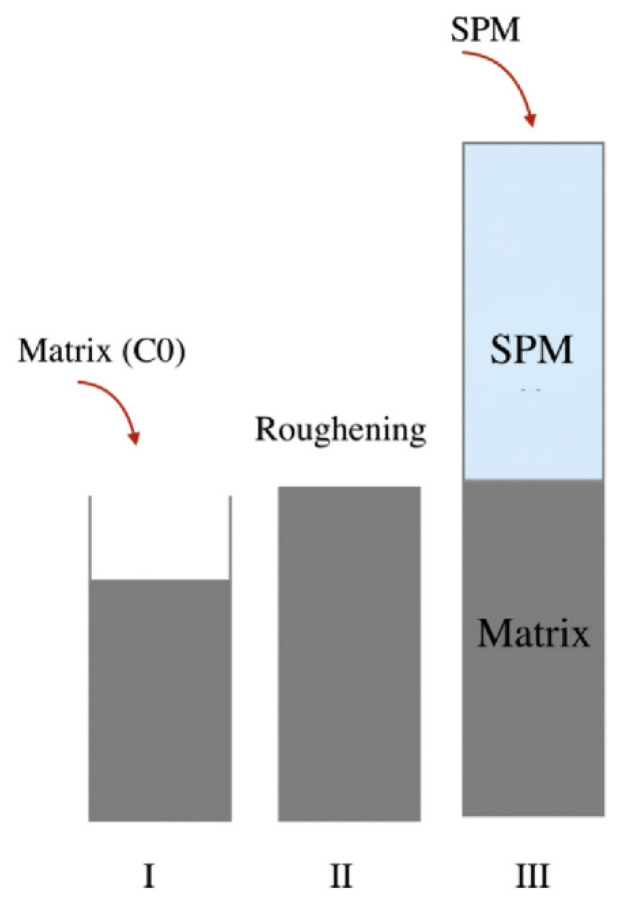
Casting procedure of mortar for the measurement of viscous performance [74].

**Figure 16:**
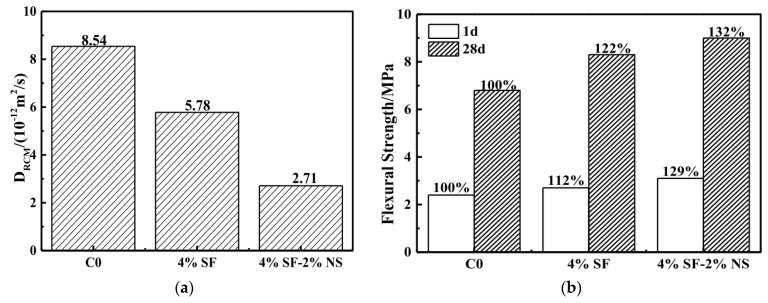
(**a**) Chloride diffusion coefficient of concrete coated by SPM for 28 days; (**b**) interfacial bond strength of matrix and SPM [74].

To enhance the penetration resistance of the whole system under a corrosive environment, Zhang et al. [74] tried to use nanosilica (NS) and silica fume (SF) to modify cement mortar as a surface protection material (SPM). Eight different coating compositions were made by varying the proportions of silica fume and nanosilica. After casting reference mortar, at the time between the initial and final setting, the surface of the sample was roughened to increase the contact area between the surface and the SPM. Then, a 5 mm thick coating was applied to the concrete sample, and after a 28-day curing period, a rapid chloride migration test was performed on the coated structure. These coated structures were used for the measurement of rapid chloride migration to check the impermeability of mortar with SPM. Additionally, to characterize the interfacial bond strength between the matrix and the SPM, the flexural strength of mortar prepared by twice-casting was measured (Figure 15). 4% SF reduced the chloride diffusion coefficient by 32.32% in comparison with reference concrete, however, the addition of 2% NS in 4% SF reduced the coefficient by 68.27% compared to a reference, indicating excellent resistance to chloride penetration (Figure 16a). Along with the improved penetration resistance, coated sample 4%SF2%NF showed increases in flexural strength of 29% and 32% after 1 and 28 days, respectively (Figure 16b). Therefore, the authors found the coating materials performed successfully in corrosive environments, as coated samples showed significantly increased compressive strength and impermeability by densifying the interfacial transition zone (ITZ) and refining the pore structure. Additionally, coated samples showed better dimensional stability with lower shrinkage compared with reference mortar.

Lavigne et al. [75] developed an innovative approach to simulate the biodeterioration of industrial cementitious products in sewer environments and validated it using BFSC (blast furnace slag cement) and CAC (calcium aluminate cement) linings. These two coatings were applied to the pipes and were kept exposed for 107 days in biogenic acid concrete (BAC) test setups. The performance was evaluated with a scanning electron microscope (SEM) coupled with energy dispersive X-ray spectrometry (EDS), electron probe microanalysis (EPMA), and X-ray diffraction (XRD). Due to the precipitation of secondary ettringite, abundant cracking was observed in BFSC lining, whereas no cracking was observed in CAC lining. The degraded layer depths of BSFC and CAC linings were 700 μm and 150 μm, respectively. Similar results were obtained by [76,77]. 

Various researchers highlighted the potential of geopolymer technology for wastewater applications. Because of geopolymers’ characteristics, geopolymer concretes (GPC) combine the desirable properties of vitreous ceramic pipes (permeability, acid, and abrasion resistance) with the advanced performance of concrete pipes (any diameter pipe possible, no-dig repair, and low-temperature molding), but at the same time overcome the individual limitations of both (low durability, higher cost, brittleness, small diameter) [14]. According to recent publications [78,79], GPC exhibits higher acid resistance compared to existing concepts of concrete durability using a sacrificial layer. Today, all cement-based products and alkali-activated Ca-rich binders contain Ca-rich acid-dissolvable products, which is avoided in GPC technology, thereby increasing acid resistance [80]. 

Other than the abovementioned coating materials used for the protection of sewage pipes from corrosion are PVC liners, coal tar coatings [80], epoxy, acrylic resins, polyester-based polymers [49,68,81,82], and high-density polyethylene (HDPE) liners [83,84,85]. It was also reported that silanes can be used for significantly reducing chloride penetration. According to [86], silica-based hybrid nanocomposite can be used as surface treatment of hardened cement-based materials, which can significantly lower water absorption rate and gas permeability coefficient. The authors of [87] concluded that the chemical and thermal stability, micropore structure, and antimicrobial characteristics of processed or natural zeolites make them efficient protective coating materials against bacterially induced corrosion.

Along with these coating materials, recently, studies have been carried out using alkali-activated material, nanomaterial, nitrite spray, and CAC (calcium aluminate cement)-GGBFS (ground granulated blast furnace slag) blended with SHCC (strain-hardening cementitious composite). The authors of [88] studied the application potential of alkali-activated concrete (AAC) against MICC; analyzed its long-term bacteriostatic performance, acid resistance, and impermeability of alkali-activated concrete; and compared the results with normal concrete. Positive results in terms of physical resistance, such as prevention of microbial growth, bacterial inhibition, and refinement of pore structure to block corrosive media infiltration; chemical resistance, such as excellent acid resistance and resistance to sulfate attack; and long-lasting bacteriostatic performance were observed. The authors of [89] discussed a methodology which can prevent MICC and pointed out that nanomaterials can hinder the biodeterioration of concrete, while [90] studied the ability of nitrite spray to mitigate corrosion on corroded concrete as well as re-establishment in a real sewer system. They found that the corrosion rate of concrete was reduced by 40–90% for 6 months by a single nitrite spray, whereas, the biannual application of nitrite spray was able to achieve a 1.6–10 times extension of sewer service life with a fairly low cost. The work presented in [91] was a pilot study for the structural performance of damaged reinforced concrete pipes (RCP) retrofitted by CAC-GGBFS blended with SHCC lining, and the experimental results showed it as an effective repair solution to damaged concrete sewerage pipelines, as it showed a 50.6% increase in ultimate load-carrying capacity compared to original RCP.

However, the efficiency of coatings is achieved only if high quality of application (workmanship) and a completely sealed system are achieved [92]. The likelihood of debonding is higher behind the coatings due to the building of hydraulic pressure [93,94]. Sometimes, to increase the robustness of the design, a two-barrier system, i.e., one layer of coating and another layer of sacrificing concrete, is adopted [83,93,95] A summary of the various coatings used to reduce MICC is presented in Table 3.

## 6. Conclusions and Future Perspectives

Massive sewer systems have been built using concrete as the parent material. Though concrete is one of the most suitable construction materials in most areas, it has limitations in severe environments, such as sewerage systems. Global sewer systems are facing one of the most serious and costly problems due to microbiologically induced concrete corrosion (MICC). Substantial research has been carried out in this area for a significant amount of time; still, the impact of research findings on real construction practice is limited. The present study reviews the various elements of MICC, especially in the sewer environment. It focuses mainly on aspects such as the mechanism and process of microbial deterioration, methods to study MICC, effects of biocorrosion on concrete properties, and various coating materials tested to mitigate biocorrosion.

- Due to the lack of standardized testing methods, various researchers have developed different methods to study biocorrosion. Therefore, it is difficult to compare the test procedures and results obtained with various methods, demanding an urgent need to develop standard testing methods and acceleration procedures by considering all the aspects of MICC.
- A clear relation between the corrosion behavior (corrosion rate) obtained in the laboratory tests and that from the site is still not well established. To better understand the corrosion behavior, there is an urgent need to develop quantitative models which can accurately predict each MICC process.
- More investigations are required to understand the microbial activities throughout different stages of the MICC process.
- Other areas which require attention for further studies could be the rate-limiting factors for microbial activities at different stages, the roles of different bacteria species at each stage of the corrosion processes, the role of the corrosion layer as a growth matrix and food provider for bacteria, and the distribution of different bacteria species within corrosion layer.
- Lastly, the effectiveness and applicability of the coating materials, such as polyurethane, cement, geopolymer, a blended mix of geopolymer and magnesium phosphate, resin powder with (PVA), nylon fibers, silica fume, nanosilica, BFSC, and CAC, are discussed in detail. Although some of these materials provide significant improvements in concretes performance against biocorrosion, attention should be given to developing novel sustainable materials which can entirely withstand extremely aggressive and corrosive sewer environments.

## Figures and Tables

**Table 1:** Summary of chemical tests performed in various studies.

Sr. No.	Specimen	Concentration of Sulfuric Acid	Duration of Immersion	Reference
1.	Mortar and concrete, 100 mm cubes	2% (pH-1.78)	1 to 32 days	[47]
2.	Concrete, inner tank with diameter 0.9 m and outer tank with diameter of 1.2 m	10%	42 to 56 days	[48]
3.	Concrete, cylinders with 76 mm diameter and 152 mm height	3% (pH-0.45)	7 days	[49]
4.	Concrete, prisms with dimensions 38 × 38 × 200 mm	pH-0.5 to 2	7 to 112 days	[50]
5.	Mortar; 50 mm cubes and 25 × 25 × 250 mm mortar bars	1.5% (pH~1.1)	6 months	[42]
6.	Mortar; cylinder with inner diameter 50 mm, total diameter 100 mm, and height 50 mm	10%	7 days	[39]

**Table 2:** Summary of effect of biocorrosion on strength loss.

No.	Specimens	Exposure	Parameter	Result	Reference
1.	Concrete, cylinders with a diameter of 75 mm and height of 150 mm	Sulfuric acid immersion (5%; 12 weeks)	Compressive strength	Decreased up to 34%	[66]
2.	Mortar, prisms with dimensions 40 × 40 × 160 mm	Sulfuric acid immersion (pH-2; 90 days	Compressive strength	Reduced by 50%	[5]
3.	Concrete, 150 × 150 × 150 mm cubes	In situ test (6, 12, 18 months)	Compressive strength	Increased by 68% and 17% after 12 and 18months, resp.	[65]
4.	Concrete, prisms with dimensions 20 × 20 × 100 mm	Biosulfuric acid immersion (9 g/L; 12 months)	Flexural & Compressive strength	Flexural and compressive strength were reduced by an average of 40% & 20% respectively	[67]
5.	Concrete, prisms with dimensions 38 × 38 × 200 mm	Sulfuric acid immersion (pH-0.5; 7 112 days)	Relative Dynamic Elastic Modulus	Decrease from 100 to 65% average	[50]
6.	Mortar, arch-shaped	Accelerated biocorrosion chamber (6 months)	Flexural Strength	Decrease by 73%	[1]
7.	Mortar, 50 mm cubes and 25 × 25 × 250 mm mortar bars	Sulfuric acid immersion (1.5%; pH~1.1; 6 months)	Compressive strength	Decrease of 43.3 to 67.6%	[64]

**Table 3:** Summary of the various coating materials used to reduce MICC.

Coating Material	MICC Method	Performance Evaluation	Conclusion	Reference
Polyurethane-1 Polyurethane-2	Sulfuric acid Immersion	Hydrostatic test Bonding strength Pinhole test—chemical resistance	No failure in either coating after 5 years of exposure	[49]
Cement Mortar Geopolymer Blended mix of geopolymer and magnesium phosphate	Accelerated biocorrosion chamber	pH variations Strength loss Surface morphology Pull-off test	Geopolymer coating showed best results for virgin as well as corroded pipes following blended coating.	[1]
Resin powder (RP) composed of polyvinyl acetate (PVA) Nylon fibers (NF)	Sulfuric acid Immersion	Compressive strength Setting time Water-tightness test Sulfur resistance test Freeze-thaw cycle test	For moderate environmental conditions, 4.5% resin powder coating without fiber showed the best results, and for severe conditions, a combination of RP and NF was recommended	[39]
Silica fume (SF) Silica fume and nanosilica-modified cement mortar (SF & NS)	Sulfuric acid Immersion	Compressive strength Flexural strength Rapid chloride migration Shrinkage Hydration heat Porosity	Coated samples significantly increased compressive strength and impermeability by densifying interfacial transition zone (ITZ) and refining pore structure along with better dimensional stability and less shrinkage compared with reference mortar.	[74]
Blast furnace slag cement (BFSC) Calcium aluminate cement (CAC)	Biogenic Acid Concrete (BAC) setup	Scanning electron microscope (SEM) coupled with energy dispersive X-ray spectrometry (EDS) Electron probe microanalysis (EPMA) X-ray diffraction (XRD).	CAC lining showed no cracking, whereas BFSC showed abundant cracking due to precipitation of secondary ettringite.	[96]
